# Freiburg Neuropathology Case Conference: 68-Year-Old Patient with Slurred Speech, Double Vision, and Increasing Gait Disturbance

**DOI:** 10.1007/s00062-024-01385-4

**Published:** 2024-02-12

**Authors:** C. Zander, M. Diebold, M. J. Shah, B. Malzkorn, M. Prinz, H. Urbach, D. Erny, C. A. Taschner

**Affiliations:** 1 Departments of Neuroradiology, University of Freiburg, Freiburg, Germany; 2 Neuropathology, University of Freiburg, Freiburg, Germany; 3 Neurosurgery, University of Freiburg, Freiburg, Germany; 4 Medical Centre—University of Freiburg, Faculty of Medicine, University of Freiburg, Breisacherstraße 64, 79106 Freiburg, Germany; 5 Institute of Neuropathology, University Hospital Düsseldorf, Düsseldorf, Germany

**Keywords:** Brain metastasis, Hemangioblastoma, Adult medulloblastoma, Pilocytic astrocytoma, High-grade astrocytoma with piloid features (HGAP)

## Case Report

**Fig. 1:**
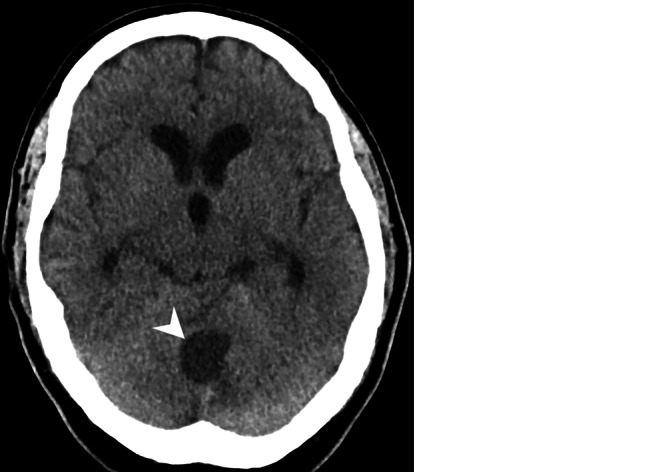
Axial CT image of the head in soft tissue settings obtained upon admission showed a cystic lesion within the cerebellum (*arrowhead*). Note the distended lateral and third ventricles, indicating obstructive hydrocephalus

**Fig. 2:**
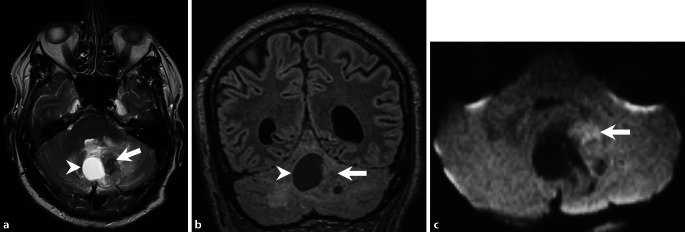
Axial T2 weighted images (**a**) and coronal fluid attenuated inversion recovery (FLAIR) images (**b**) confirmed a space-occupying intra-axial cerebellar lesion. The lesion comprised a sizable cystic portion (**a** + **b**, *arrowhead*) adjacent to a solid tumor component (**a** + **b**, *arrow*). Axial diffusion-weighted MR images (b-value: 1000) indicated restricted diffusion in parts of the solid tumor component (**c**, *arrow*)

**Fig. 3:**
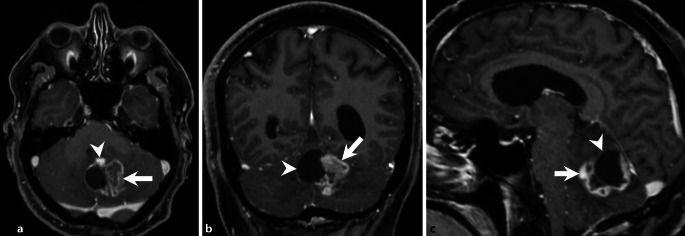
Axial (**a**), corona (**b**), and sagittal (**c**) T1-weighted images, acquired post-gadolinium (Gd) administration, revealed a capsular enhancement pattern in the cystic portion (**a**–**c**, arrowhead), while the solid tumor portion displayed a heterogeneous contrast enhancement (**a**–**c**, arrow)

A 68-year-old male patient had been experiencing double vision and dizziness for approximately 14 days. He presented with an increasing gait disturbance and slurred speech. Subsequent computed tomography (CT) and magnetic resonance imaging (MRI) scans revealed an infratentorial cystic mass lesion (Figs. 1, 2 and 3). Due to its space-occupying effect on the 4th ventricle and the resulting hydrocephalus, surgery was urgently indicated.

The operation was performed with the patient under general anesthesia and in the prone position. Following a suboccipital craniotomy and corticotomy, the well-defined tumor was circumferentially removed. The cyst contents were xanthochromic, and the cyst capsule was firm. The tumor exhibited inhomogeneity with both hard and soft parts, partly fibrotic, partly soft. From a surgical perspective, the tumor was more characteristic of a low-grade glioma. Postoperatively, the patient was gradually mobilized, without any additional neurological deficits, and postoperative adjuvant radiotherapy was subsequently initiated.

## Imaging

Upon admission, native cranial computed tomography (CT) revealed a cystic lesion within the cerebellum (Fig. 1, *arrowhead*). Note the distended lateral and third ventricles indicating obstructive hydrocephalus. Subsequent magnetic resonance (MR) imaging confirmed a space-occupying intra-axial cerebellar lesion (Figs. 2 and 3).

On T2-weighted images, the lesion comprised a sizable cystic portion (Fig. 2a, b, *arrowhead*) adjacent to a solid tumor component (Fig. 2a, b, *arrow*). Diffusion-weighted MR images (b-value: 1000) indicated restricted diffusion in parts of the solid tumor component (Fig. 2c, *arrow*). T1-weighted images, acquired post-gadolinium (Gd) administration, revealed a capsular enhancement pattern in the cystic portion (Fig. 3a–c, *arrowhead*), while the solid tumor portion displayed a heterogeneous contrast enhancement (Fig. 3a–c, *arrow*).

## Differential Diagnosis

Space-occupying lesions in the posterior fossa usually manifest with similar symptoms. These include, for example, headaches, dizziness and hearing loss [1]. In the case of compression of the fourth ventricle, obstructive hydrocephalus with signs of increased intracranial pressure may occur [1]. In children, the posterior fossa is a very common predilection site for tumors [1]. Besides neoplasia patients may present with vascular lesions (e.g., hemorrhage or vascular malformation), (congenital) cysts or infectious pathologies such as an abscess [1].

### Brain Metastasis

Brain metastases represent the most common malignant central nervous system (CNS) tumors in adults with lung cancer, breast cancer and melanoma being the most frequent primary malignancies [2–4]. The most common localization in the brain parenchyma is juxtacortical and in the border zone areas [5]. Even though only 15% of metastases are cerebellar, they are still the most common malignant entity when a tumor is found in the posterior fossa [5, 6]. On MRI, metastases are typically isointense to hypointense in T1, while they show a variable signal in T2 [7]. A ring-shaped contrast enhancement is common, especially among larger lesions [7]. The signal intensity on diffusion-weighted imaging can vary depending on different histology and thus cell density [8]. CT is clearly less sensitive than MRI in the detection of metastases, but it is very well-suited as an initial examination in everyday clinical practice to rule out potentially life-threatening complications [9]. CT may show metastases as hypodense, isodense or hyperdense to the brain parenchyma with varying degrees of perifocal edema [5, 7]. Especially in the context of hemorrhage the radiological appearance on CT and MRI scans may vary [7]. The majority of patients present with more than one metastasis [4, 5].

Due to the relatively high incidence among adults and the variety of possible presentations on imaging, metastases are an important differential diagnosis in this case. Even if no primary tumor is known yet, metastases should be included in the clinical consideration [10].

### Hemangioblastoma

Hemangioblastomas, a low-grade lesion WHO grade 1, are typically associated with von Hippel-Lindau disease, but can also occur sporadically [11]. These vascularized tumors occur either with associated cysts (which can be intratumoral and/or peritumoral) or without a cystic component [6]. On MRI the solid proportion mostly presents with contrast enhancement and may show flow voids on T1w and T2w [12]. It is notable that symptoms usually occur due to the growth of the cystic component, especially within lesions with a peritumoral cyst [6, 11]. In contrast to the intramural cyst, which can be seen as the result of tumor necrosis, the peritumoral cysts develop due to increased interstitial pressure and vascular permeability [6]. As the diameter increases, the lesions also have an increased risk of spontaneous hemorrhage [13].

The most common localization is the posterior cranial fossa (about 95%) [12]. Of all brain tumors in the posterior fossa among adults, they account for up to 10% [6]. Our case shows a tumor with a cystic and solid component in the typical localization. Nevertheless, the wall enhancement described in our case is rather atypical for hemangioblastomas [12]. Also, there was no known von Hippel-Lindau disease; however, the sporadically occurring hemangioblastomas are more likely to be found in adults, which would fit our case [6].

### Adult Medulloblastoma

A rare but also possible diagnosis is the adult medulloblastoma. Medulloblastomas are the most common tumors in children with a proportion of approximately 30%, whereas these account for only 1–3% of all adult primary brain tumors [6]. Clinical manifestations of the tumor usually include increased intracranial pressure and symptoms such as headaches, dizziness and ataxia [14]. Adult medulloblastomas have some special features that distinguish them from lesions in younger patients: In older patients medulloblastomas typically arise in the cerebellar hemisphere, whereas they mostly originate from the vermis in children [6, 14]. While medulloblastomas have a quite good prognosis in younger patients (if total resection is obtained) among adults they seem to have a lifelong risk for recurrence [6, 15]. On MRI, adult medulloblastomas appear hyperintense in T1w and hypointense in T2w [14].

The medulloblastoma can be divided into four molecular subgroups (WNT, SHH, group 3 and group 4) with each of them having different characteristics [16]. Especially the WNT and SHH subtype show similarities in MRI to our case with a high incidence of cysts and perifocal edema as well as heterogeneous contrast enhancement. However, our case differs by a lack of signal increase on diffusion-weighted imaging (which is typical for medulloblastomas), although a signal decrease is seen in the ADC map [14].

### Pilocytic Astrocytoma

One diagnosis that quickly comes to mind is pilocytic astrocytoma. These WHO grade 1 tumors usually present with a large cystic component and a smaller nodule [17]. On CT this is seen as a hypodense to isodense lesion with only little or no edema, in some cases with associated calcifications [18]. MRI shows a T1w hypointense to isointense tumor which presents as hyperintense in T2 with a strong contrast enhancement of the node while enhancement of the cystic wall is usually less common [17, 18]. Sporadic astrocytomas are usually found in the posterior fossa [17]; however, these tumors typically occur in younger patients where they account for up to 40% of all brain tumors [17, 19]. In this population, the tumors have a very good outcome with a survival rate of over 95%, whereas the prognosis is significantly worse in older patients [20].

Although the tumor with cystic and solid components described in our case initially suggests a pilocytic astrocytoma, there are several features that do not fit: the cerebellar localization is rather untypical at this age, as most pilocytic astrocytomas in adults are found supratentorially [19, 20]. In addition, our case shows a clear perifocal edema, which rather suggests a higher grade tumor. In particular, the configuration of the solid component, which, although showing strong contrast enhancement at the margins, appears necrotic in the center, draws attention to a different entity.

### High-grade Astrocytoma with Piloid Features (HGAP)

HGAP is a novel tumor entity, first listed in the 2021 WHO classification of tumors of the CNS which is defined by a specific methylation profile [21, 22]. The histopathology of this entity shows some similarities with higher grade gliomas, such as glioblastoma [23]. To date there are only few reported cases [21]. Most of the patients appear to be middle-aged [23]. There also may be an association with neurofibromatosis type 1 [21]. Due to the small number of cases it is difficult to name a clear distribution, but there is presumably a preference for the posterior fossa, especially the cerebellum [21]. The clinical outcome is poor [21, 23].

HGAP usually presents as T1w hypointense to isointense with inhomogeneous contrast enhancement and hyperintense signal in T2w [21]. As these tumors are IDH-wildtype, a T2/FLAIR mismatch is not to be expected [24].

The radiographic and clinical features of this entity fit our case well, which is why HGAP is an important differential diagnosis. Ignoring the localization in the posterior fossa, the MRI in our case could also lead to the suspicion of a glioblastoma, which also illustrates the similarity of the HGAP to higher grade gliomas in imaging; however, due to the cerebellar occurrence of our tumor, glioblastoma was not addressed separately as a differential diagnosis in this case [6].

## Histology and Immunohistochemistry

**Fig. 4:**
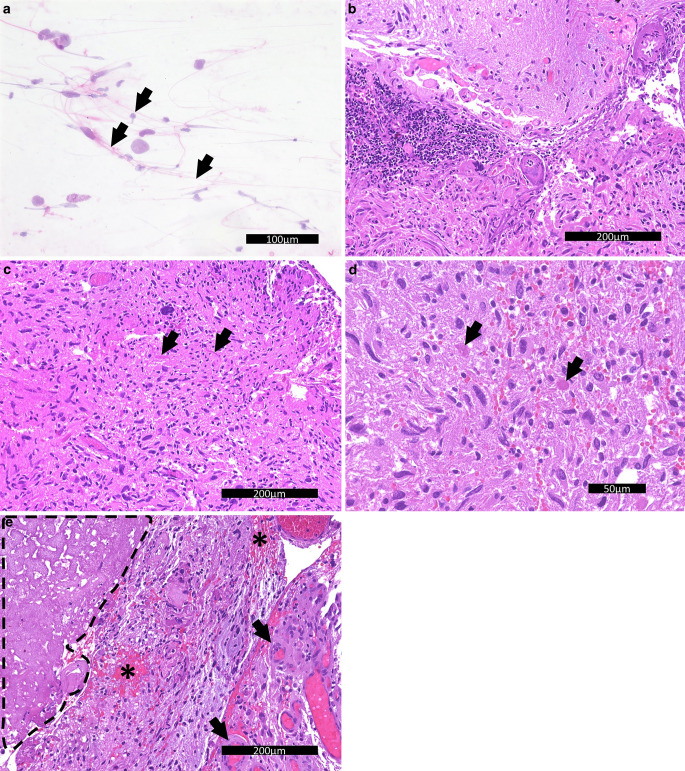
Hematoxylin-eosin (H&E) stained smear (**a**) of the initial intraoperative sample showed pilocytic extensions. Scale bar: 100 µm. Hematoxylin-eosin (H&E) stained sections showed (**b**): different growth patterns of tumor tissue and lymphocytic infiltrations. Scale bar: 200 µm, **c** Rosenthal’s fibers (*arrows*). Scale bar: 200 µm, **d** granular bodies (arrows). Scale bar: 50 µm, and **e** necrotic tissue (circumscribed area), fresh bleeding (*) and vascular proliferates (*arrows*). Scale bar: 200 µm

On biopsy of the lesion, pleomorphic glial tumor cells could be identified in the initial intraoperative smear. Pilocytic extensions appeared to characterize these tumor cells (Fig. 4a). The formalin-fixed and paraffin-embedded preparation of the specimen shows a moderately cell-rich, pleomorphic, astrocytically differentiated glioma with regional biphasic growth pattern, in the hematoxylin and eosin (H&E) stain (Fig. 4b). The majority of the tumor cells exhibit elongated nuclei with finely dispersed chromatin and form a spindle cell cytoplasm. Focally, eosinophilic swellings reminiscent of Rosenthal’s fibers (Fig. 4c), granular bodies (Fig. 4d) and disseminated protein droplets can be seen. Moreover, large areas of necrosis and fresh hemorrhages, and focal pathological vascular proliferations can be noted (Fig. 4e). At the tumor edge, incisions of cerebellar cortex with a relatively sharp border to the tumor tissue are found. In several locations, lymphocyctic infiltrates are recognized perivascularly (Fig. 4b).

**Fig. 5:**
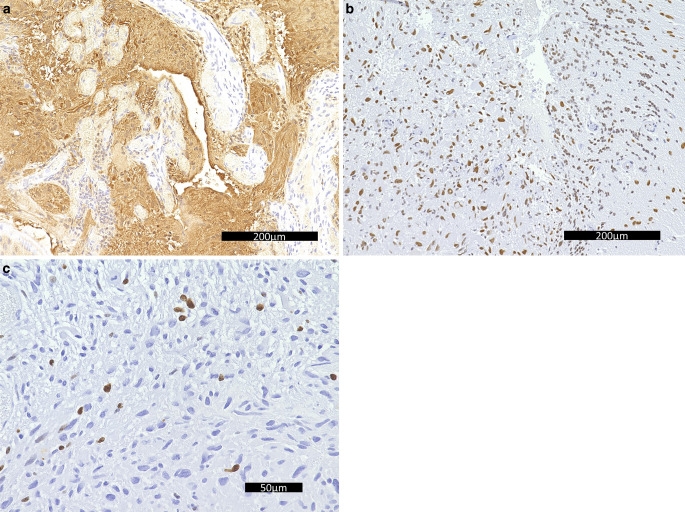
Immunohistochemistry of the lesion for GFAP (**a**) showed positive tumor cells. Scale bar: 200 µm, for ATRX (**b**) showed retained signal in tumor cells. Scale bar: 200 µm, and for MIB1/Ki-67 (**c**) showed increased proliferation. Scale bar: 50 µm

Further work-up included immunohistochemistry and molecular pathology. On immune labelling, the tumor cells reacted partially positive for the glial fibrillary acidic protein (GFAP, Fig. 5a) and, less frequently, the oligodendrocyte transcription factor Olig2 (not shown). The nuclear expression of ATRX is preserved in tumor cells (Fig. 5b). Endothelial cells, but not tumir cells, show positive reaction for CD34 (not shown). In the tumir area, the reaction for synaptophysin is negative indicating no diffuse infiltration (not shown). The proliferation marker MIB‑1 focally marks more than 10% of the tumir cells positively indicating significantly increased proliferation (Fig. 5c). The reaction for the histone H3 K27M mutation is negative (not shown). Moreover, no loss of H3 K27 trimethylation or overexpression of the EZH1/2 inhibitory protein EZHIP is detectable (not shown).

**Fig. 6:**
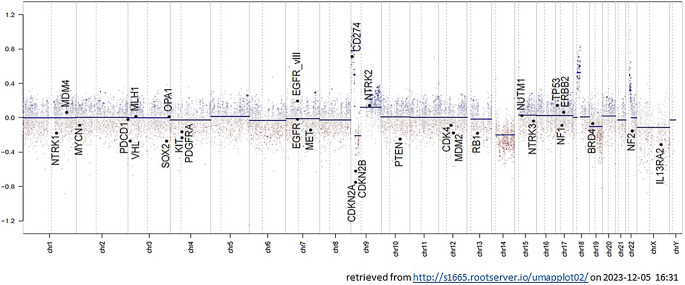
Results of DNA methylation profile obtained via EPIC array showing copy number variation profile, as obtained from EpiDiP (http://s1665.rootserver.io/umapplot02/)

No IDH1/2 mutations were identified by immunohistochemistry or sequencing. Additional next generation sequencing was carried out at the Institute of Neuropathology Düsseldorf after joint evaluation of the case. Hereby, a supposedly pathological variant in neurofibromatosis 1 (NF1: NM_001042492.3: exon21: c.2672_2673delTC: pVal891GlufsTer14) was detected with suggested loss of function. Wild-type sequences were detected regarding H3-3A, IDH1, IDH2, BRAF and TERT. A BRAF duplication could not be detected. Additionally, an RNA fusion analysis was also carried out with no relevant fusions detected, in particular no fusion involving BRAF. Finally, the DNA methylation profile was generated from a vital, tumor-cell rich area, analyzed and compared with the methylation patterns of reference cases via the molecular neuropathology web tool (https://www.molecularneuropathology.org/mnp, [25]). Various classifiers were used as a reference database, none of which resulted in a formal, significant match (brain tumor classifiers, Versions: 12.8, 12.5, and 11.4, additional analysis by EpiDiP-database (https://epidip.org, [26])). The copy number variation profile revealed only circumscribed gains on chromosomes 9, 18 and 22, and losses on chromosomes 9 and 14, including a relative loss of CDKN2A/B (Fig. 6). Independent analyses by MLPA and droplet digital polymerase chain reaction verified a reduced gene dose of CDKN2A/B, but did not suggest homozygous loss.

## Diagnosis

### High-grade Astrocytic Glioma, not Elsewhere Classified (NEC)

The specimen of this cerebellar tumor shows an astrocytic IDH-wildtype glioma with signs of anaplasia and increased proliferation. Despite its extensive characterization, this tumor’s definite assignment to one tumor type defined by the current WHO classification [27] remains difficult. The differential diagnosis of this tumor comprises glioblastoma CNS-WHO grade 4, diffuse mid-line gliomas CNS-WHO grade 4, pilocytic astrocytoma CNS-WHO grade 1, and high-grade astrocytoma with piloid features (no CNS-WHO grade assigned yet).

Assessing an IDH-wildtype tumor with necrotic areas and endothelial proliferates, glioblastoma needs to be discussed as a differential diagnosis. In the methylation array no match for glioblastoma and no corresponding gains on chromosome 7 or losses on chromosome 10 in the copy number profile (Fig. 6) were detected. Localization in the cerebellum is relatively rare in glioblastoma [28]. Moreover, a solid growth pattern of the tumor and also the copy number profile would be rather unusual for a cerebellar glioblastoma, IDH wild-type.

In contrast to glioblastoma, the tumor location, combined with features of a higher grade tumor, could point towards the diagnosis of a diffuse midline glioma. This entity, however, was excluded by absence of H3K28M (H2K27M) mutation, EZHIP overexpression, and retained H3K28me3 expression and does not usually display the solid growth pattern of the tumor assessed here [29].

The relatively solid growth and the piloid differentiation features would be consistent with a high-grade astrocytic glioma with piloid features, although the methylome analysis did not show assignment to the corresponding DNA methylation profile. Moreover, the NF1 variant may argue in favor of the presence of HGAP [24], while NF1 variants can also be found in glioblastomas and pilocytic astrocytomas, among others [30]. HGAP characteristically occurs in middle-aged patients in the area of the cerebellum [23, 27]. The current WHO classification of brain tumors stipulates a suggestive DNA methylation profile for diagnosis or exclusion. As, in the case discussed here, HGAP was identified by the recent classifiers at a very low calibrated score rendering the formal diagnosis of HGAP impossible [27].

A pilocytic astrocytoma with signs of anaplasia may also be considered in the differential diagnosis of HGAP, as no clear-cut match is retrieved from the methylation pattern [27]. Anaplastic pilocytic astrocytomas, the diagnostic umbrella term used in previous WHO classifications for CNS tumors [23], now largely absorbed by the newly defined diagnosis of HGAP, have been reported to not fulfil the criteria of HGAP in approximately 20% of cases with still poorly defined clinical and prognostic outcomes. In about one third of cases of pilocytic astrocytomas with signs of anaplasia, germline or somatic NF1 mutations can be detected [27]; however, formal criteria for this subset of pilocytic astrocytomas or a typical methylation profile have not been defined and clinical information is sparse, but suggestive of substantially more aggressive behavior in most cases [23, 31, 32]. Therefore, assignment of the current case to the formal diagnosis of pilocytic astrocytoma is complicated by the absent loss of ATRX and remains disputable [27].

In summary, the present case cannot, despite thorough pathological workup, be assigned to one established tumor type in the current WHO classification of CNS tumors: it does not show the diffuse growth of glioblastoma or diffuse midline gliomaor exhibit the classical methylation pattern of HGAP or pilocytic astrocytoma. The current classification system allows descriptive diagnoses labelled by the designation “not elsewhere classified (NEC)”, when full workup according to guidelines has been performed, but does not match with diagnostic features of established entities [27]. The presented case was discussed between two centers of neuropathology finally resulting in the concordant diagnosis of a cerebellar high-grade astrocytic glioma, not elsewhere classified (NEC). This NEC diagnosis illustrates the limited prognostic estimation that can be provided for this case, similar to the differential diagnoses of the newly defined HGAP and the loosely defined subset of pilocytic astrocytoma cases with signs of anaplasia.
